# Genetic Structure of the Endangered Coral Cladocora caespitosa Matches the Main Bioregions of the Mediterranean Sea

**DOI:** 10.3389/fgene.2022.889672

**Published:** 2022-07-26

**Authors:** Mar Repullés, Violeta López-Márquez, José Templado, Marco Taviani, Annie Machordom

**Affiliations:** ^1^ Department Biodiversidad y Biología Evolutiva, Museo Nacional de Ciencias Naturales, MNCN (CSIC), Madrid, Spain; ^2^ ISMAR-CNR, Istituto di Scienze Marine, Consiglio Nazionale delle Ricerche, Bologna, Italy; ^3^ Stazione Zoologica Anton Dohrn, Naples, Italy

**Keywords:** asexual reproduction, *Cladocora caespitosa*, clones, dispersion, marine connectivity, microsatellites, population structure

## Abstract

Population connectivity studies are a useful tool for species management and conservation planning, particular of highly threatened or endangered species. Here, we evaluated the genetic structure and connectivity pattern of the endangered coral *Cladocora caespitosa* across its entire distribution range in the Mediterranean Sea. Additionally, we examined the relative importance of sexual and asexual reproduction in the studied populations and their genetic diversity. A total of 541 individuals from 20 localities were sampled and analysed with 19 polymorphic microsatellite markers. Of the genotyped individuals, 482 (89%) had unique multilocus genotypes. Clonality percentages of the populations varied from 0% (in eight populations) to nearly 69% (in one population from Crete). A heterozygosity deficit and a high degree of inbreeding was the general trend in our data set. Population differentiation in *C. caespitosa* was characterised by significant pairwise *F*_*ST*_ values with lower ones observed at an intraregional scale and higher ones, between populations from different biogeographic regions. Genetic structure analyses showed that the populations are divided according to the three main sub-basins of the Mediterranean Sea: the Western (Balearic, Ligurian and Tyrrhenian seas), the Central (Adriatic and Ionian seas) and the Eastern (Levantine and Aegean seas), coinciding with previously described gene flow barriers. However, the three easternmost populations were also clearly separated from one another, and a substructure was observed for the other studied areas. An isolation-by-distance pattern was found among, but not within, the three main population groups. This substructure is mediated mainly by dispersal along the coastline and some resistance to larval movement through the open sea. Despite the low dispersal ability and high self-recruitment rate of *C. caespitosa*, casual dispersive events between regions seem to be enough to maintain the species’ considerable genetic diversity. Understanding the population connectivity and structure of this endangered scleractinian coral allows for more informed conservation decision making.

## Introduction

Marine population dynamics is determined by connectivity among populations, which can play a key role in species resilience (Cowen and Sponaugle, 2009). Gene flow between geographically separated populations of marine species is mainly governed by both the biological traits of species and oceanographic dynamics (Villamor et al., 2014). Historical barriers, currents, habitat discontinuities and shoreline configuration, along with the larval life span and behaviour of a species, can all drive genetic differentiation between populations (González-Wangüemert et al., 2010). For instance, in the Mediterranean Sea, several currents and fronts have been described as potential barriers to gene flow (Coll et al., 2010; Villamor et al., 2014; Pascual et al., 2017), which could influence the genetic structure of species. Larval dispersal capacity, settlement success and survival of newly settled post-metamorphic individuals until recruitment are also crucial factors affecting species distribution and population connectivity (Botsford et al., 2001; Pascual et al., 2017). Population genetics studies are essential to estimate the degree of connectivity and to identify associated processes that may influence it, such as barriers to gene flow, self-recruitment and population isolation or fragmentation, among others.

In this study, we examine the genetic connectivity of populations of *Cladocora caespitosa* (Linnaeus, 1767), a Mediterranean colonial and zooxanthellate scleractinian coral whose features are similar to typical tropical reef-building corals. This species is, for instance, able to form extensive bioherms that may fuse in reef-like structures (Kružić et al., 2008). Records show that *Cladocora* has been present in the Mediterranean basin since the Miocene (Vertino et al., 2014). Although morphologically indistinguishable from other *Cladocora* fossils, *C. caespitosa* is thought to have been present in the basin since, at least, the warm-temperate late Pliocene (Aguirre and Jiménez, 1998; Peirano et al., 2009; Bosio et al., 2021), and was particularly common in the late Pleistocene (Cuerda et al., 1986; Bernasconi et al., 1997; Amorosi et al., 2014). The species is currently distributed throughout the entire Mediterranean basin as discontinuous and isolated colonies or, more rarely, as coral beds or banks (Schiller, 1993; Peirano et al., 2004; Kersting and Linares, 2012; Chefaoui et al., 2017; López-Márquez et al., 2019, 2021). Though numerous studies on the ecology and biological traits of *C. caespitosa* have been carried out (Peirano et al., 2004; Kersting et al., 2013a, 2013b, 2014a, 2014b; Rubio-Portillo et al., 2018; Pons-Fita et al., 2020, among others), genetic connectivity studies of this species are scarce. Only three studies on the genetic differentiation of the species, all conducted at a regional scale, have been published: Casado-Amezúa et al. (2014) studied the species in the western Mediterranean where they found low genetic connectivity related to sporadic dispersal events among the studied populations, almost the same results obtained in the eastern Mediterranean by López-Márquez et al. (2021). In the third study, conducted in the Adriatic Sea and adjacent Ionian, López-Márquez et al. (2019) found that the connectivity patterns were mainly driven by the shoreline configuration.

*Cladocora caespitosa* is found in a wide variety of environments, from shallow waters to about 35 m of depth (Bellan-Santini et al., 2007). It can resist strong currents but is sensitive to high wave impact (Chefaoui et al., 2017). This emblematic species displays both sexual and asexual reproduction. Asexual reproduction can occur by fragmentation or polyp removal (Kružić et al., 2008) or by asexual buds produced by polyps (Rodolfo-Metalpa et al., 2008) that generate individuals with identical multilocus genotypes (“ramets”), which, in turn, form “genets” of potentially different sizes (Baums, 2008). Sexual reproduction in *C. caespitosa* is generally seasonal and synchronous; however, differences have been observed in distinct geographic areas. In the western and eastern Mediterranean, gonochoric colonies release gametes at the end of summer, when temperatures begin to fall (Kersting et al., 2013b; Hadjioannou, 2019). By contrast, in the Adriatic Sea, hermaphroditic colonies reproduce at the beginning of the summer, when temperatures begin to rise, typically coinciding with a full moon (Kružić et al., 2008). It is unknown if these reproductive differences are associated with genetic differences.

*Cladocora caespitosa* might have been a keystone species of Mediterranean benthic communities in the past, thus playing an important role in the biodiversity of this marine realm. In fact, bioconstructions formed by this coral are known to harbour a high diversity of micro- and macrofauna (Koukouras et al., 1998; Pitacco et al., 2014). Unfortunately, like many others Mediterranean marine species (Bianchi and Morri, 2000), *C. caespitosa* is currently in alarming decline (Kersting et al., 2014b) and has been categorised as an endangered species in the IUCN Red List (Casado de Amezua et al., 2015).

The main goal of the present study is to evaluate the genetic structure and connectivity pattern of *C. caespitosa* across its entire distribution range, which has not been done to date. This will allow us to have a more complete understanding of the level of connectivity and differentiation among populations, and the potential conservation implications they may have for the species. Although local populations of marine species are generally considered demographically open (Ward et al., 1994; Hellberg, 2007), high connectivity among populations of this coral would not be expected, given that eggs are negatively buoyant (Kružić et al., 2008), limiting their planktonic dispersal and favouring local retention (Kersting et al., 2014a).

We hypothesise that *C. caespitosa* has a strong population structure with genetic differentiation between populations. We also assess the relative importance of sexual versus asexual reproduction in the studied populations, and its impact on genetic diversity as some of the populations have experienced adverse conditions that tend to increase asexual reproduction, which could influence population structure (López-Márquez et al., 2021). We hypothesise that the clonal structure of *C. caespitosa* varies across its geographic range due to the influence of various extrinsic factors affecting sexual and asexual reproductive potential.

Given the status of *C. caespitosa* as an endangered species, efficient conservation management is necessary for its long-term survival. Population connectivity studies are a useful tool for this purpose as they provide data on the resilience and sustainability of a species (Gaeta et al., 2020). By providing an overview of the connectivity of an emblematic species of the Mediterranean Sea, a key factor that must be considered for conservation plans and for decision-making on the design of marine protected areas (Holland et al., 2017), we hope to contribute to the persistence of this coral species.

## Materials and Methods

### Study Site and Sample Collection

**FIGURE 1:**
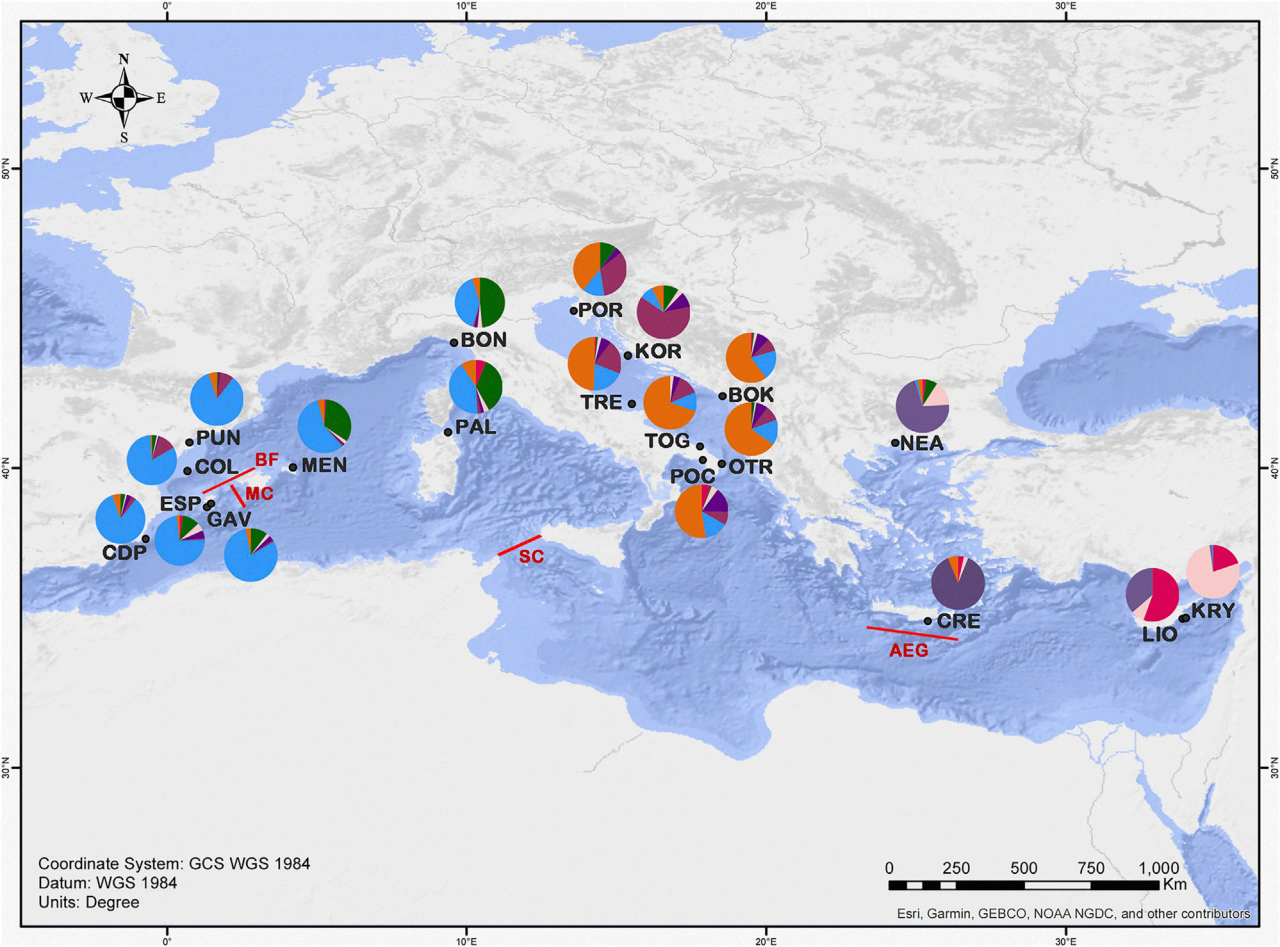
Map showing the locations of the sampled populations of *C. caespitosa* in the Mediterranean Sea. The main barriers detected in our analyses are indicated in red: MC, Mallorca Channel; BF; Balearic Front; SC, Sicily Channel and AEG, Aegeant Front (Ruiz et al., 2009; Pascual et al., 2017). Pie diagrams show a summary of the STRUCTURE results for *K* = 7 (colours as in Figure 5), in terms of the proportion of the identified genetic clusters assigned to each site.

A total of 541 individuals from 20 Mediterranean localities were collected by SCUBA diving. Due to the close proximity of Bonassola and Framura (both in the Ligurian Sea), and the relatively low number of specimens sampled there, the two localities were analysed as a single population (BON). Four localities were sampled in the eastern Mediterranean: one each in Crete and Greece and two in Cyprus. Seven localities were sampled in the central Mediterranean in the Ionian Sea or along both coasts of the Adriatic Sea in Italy, Croatia and Montenegro. Nine localities were sampled in the western Mediterranean, in Spain and western Italy (Table 1; Figure 1).

**TABLE 1:** List of the sampling localities of *C. caespitosa.* For each location, sub-basin and country are indicated in parentheses. Also provided are the population codes, GPS coordinates and number of samples (*N*) collected from each locality.

Location	Code	GPS Coordinates	*N*
Cabo de Palos, Murcia (Western, Spain)	CDP	37°37′42.90"N 0°42′7.33"O	30
Punta Gavina, Formentera (Western, Spain)	GAV	38°43′6.06"N 1°22′46.68"E	20
Isla Espardell, Formentera (Western, Spain)	ESP	38°47′16.55"N 1°28′13.02"E	31
Puerto Tofiño, Columbretes (Western, Spain)	COL	39°57′10.93"N 0°41′47.08"E	31
L’Amtella, Tarragona (Western, Spain)	PUN	40°50′26.25"N 0°44′58.92"E	30
Na Macaret, Menorca (Balearic, Spain)	MEN	40° 0′58.09"N 4°12′10.21"E	21
Palau (Tyrrhenian, Italy)	PAL	41°11"15.40"N 9°23"2.99"E	18
Bonassola (Ligurian, Italy)	BON	44°10′50.42"N 9°34′53.71"E	20
Framura (Ligurian, Italy)	BON	44°12′2.79"N 9°33′8.95"E	9
Porto Cesareo (Ionian, Italy)	POC	40°11′ 715″N 17° 55′ 077″E	35
San Foca, Otranto (Adriatic, Italy)	OTR	40° 06′ 554″N 18° 31′ 153″E	35
Torre Guaceto (Adriatic, Italy)	TOG	40° 42′ 999″N 17° 48′ 003″E	35
Tremiti Island (Adriatic, Italy)	TRE	42° 8.315′ N 15° 31.437′ E	35
Porec (Adriatic, Croatia)	POR	45°13′53.15"N 13°35′16.36"E	14
Kornati (Adriatic, Croatia)	KOR	43° 916′ 118″N 15° 146′ 881″E	35
Boka Kotorska (Adriatic, Montenegro)	BOK	42° 23′ 252″N 18° 34′ 178″E	34
Crete (Cretan, Greece)	CRE	35° 1′46.38"N 24°39′2.00"E	16
Nea Peramos (Aegean, Greece)	NEA	40° 49′31.9"N 24°20′01.9"E	31
Liopetri (Levantine, Cyprus)	LIO	34° 57′30.2"N 33°54′05.7"E	31
Kryo Nero (Levantine, Cyprus)	KRY	34° 58′57.0"N 34°01′00.8"E	30

Some of these localities were previously analysed (Casado-Amezúa et al., 2014; López-Márquez et al., 2019, 2021), however, with two different sets of primers (the first set consisted of eight primer pairs and was used by Casado-Amezúa et al., 2011 and López-Márquez et al., 2019; the second set, used by López-Márquez et al., 2021, consisted of 11 new pairs). López-Márquez et al. (2021) previously analysed three of the four eastern Mediterranean populations (NEA, KRY and LIO) using both primer sets. The results of these previous studies served as a reference and were included here in order to provide a complete picture of the species distribution across the entire Mediterranean. To facilitate comparisons, several specimens representing the analysed populations were also genotyped to ensure the uniform allele assignation with both primer sets. As the Adriatic (OTR, TOG, TRE, KOR and BOK) and Ionian (POC) populations were already analysed using the first primer set (López-Márquez et al., 2019), here we only analysed them using the second set. The newly sampled populations from the western Mediterranean (GAV, ESP, MEN, BON and PAL), the Adriatic (POR) and the eastern Mediterranean (CRE), together with the populations previously studied by Casado-Amezúa et al. (2014) (CDP, CON and PUN), were genotyped using both primer sets.

In order to avoid sampling clonal individuals, colonies collected by SCUBA diving were separated by at least 1 m. Some polyps from each colony were carefully excised and stored in vials with absolute ethanol and preserved at 4°C before processing. All necessary permits were obtained for the field studies.

### DNA Extraction and Microsatellite Amplification

The QIAGEN Biosprint 15 DNA Blood Kit (Qiagen) was used for DNA extraction and purification, following the manufacturer’s protocol. Each DNA sample was diluted to a final concentration of 0.3 ng/μl.

The DNA samples were genotyped with the 19 polymorphic microsatellite loci previously isolated for *C. caespitosa* [the eight by Casado-Amezúa et al. (2011) and the 11 by López-Márquez et al. (2021)]. The 19 primers pairs were combined in five multiplex reactions at a concentration of between 0.2 and 0.4 µM, and mixed with 1x Qiagen Multiplex PCR Master Mix, 0.3 ng of DNA and water to a total volume of 7 µl. To facilitate the genotyping, the forward primer from each pair was end-labelled with either NED, VIC, PET or 6-FAM, and the reverse primers were pig-tailed with 5′-GTTTCTT-3’ (López-Márquez et al., 2021). The PCR cycling profile included an initial denaturation step at 95°C for 5 min, followed by 35 cycles at 94°C for 30 s, 56°C for 90 s and 72°C for 30 s, and a final extension step at 72°C for 10 min.

GENEMAPPER software v4.0 (Applied Biosystems) was used to analyse the electropherograms. The presence of null alleles was checked with MICRO-CHECKER v.2.2.3 (Van Oosterhout et al., 2004).

### Genotype Analyses and Clonal Structure Parameters

By performing the option “multilocus matches” in GenAlEx 6.5 (Peakall and Smouse, 2006), we calculated the number of both unique (*N*_*g*_) and non-unique (identical) multilocus genotypes per site, which allowed us to determine the number of clones present in our data set. To analyse clonal structure parameters, and to calculate genotypic richness (standardised), genotypic evenness and genotypic diversity, we followed the methodology of Aranceta-Garza et al. (2012). Also, we classified populations according to the ratio of asexual to sexual reproduction they displayed, which was based on both genotypic evenness and genotypic diversity as in Baums et al. (2006) who delimited four groups: sexual, mostly sexual, mostly asexual and asexual. We also calculated the *D* index (Baums et al., 2006) as:

$$D=1-\left(\;\;\frac{\sum n_{i}\left(n_{i}-1\right)\;}{N\left(N-1\right)}\right)$$

Where *n*_*i*_ is the number of individuals of genotype *i,* and *N* the total number of individuals in the population. *D* values range between zero and one. If *D* equals zero, the entire population belong to the same unit of clonal growth.

### Genetic Variability

After excluding the individuals identified as clones from the data set, we used GenAlEx 6.5 and GENEPOP v4.0 (Raymond and Rousset, 1995) to calculate allelic diversity (*N*_*a*_), observed (*H*_*o*_) and expected (*H*_*e*_) heterozygosity and the index *F*_*IS*_, which is commonly used as an inbreeding coefficient, and test for Hardy-Weinberg equilibrium (HWE) and linkage disequilibrium (LD). We corrected the significance of *p* values with the sequential Bonferroni method (Rice, 1989). Prior to performing further analyses with the data set, we look for the presence of loci under selection using two methodologies: a neutrality test performed in ARLEQUIN v3.5 (Excoffier et al., 2005) and a Bayesian approach using BAYESCAN (Foll, 2012). Finally, we estimated effective population size (*N*_*e*_) with NE ESTIMATOR 2 (Do et al., 2014), following the linkage disequilibrium method (Waples and Do, 2008).

### Population Differentiation and Migration in *C. caespitosa*

To assess population structure and differentiation in *C. caespitosa*, we used various methods, including Wright’s fixation index (*F*_*ST*_), principal coordinates analysis (PCoA), isolation by distance (IBD), BARRIER analyses, the analysis of molecular variance (AMOVA), STRUCTURE and discriminant analysis of principal components (DAPC).

Population differentiation was estimated with *F*_*ST*_ between pairwise sampling sites through Weir and Cockerham’s estimators in GENETIX v.4.05 (Belkhir et al., 2004). The negative values obtained because of mathematical artefacts (*F*_*ST*_ cannot be negative) were set to zero. Standardised *F*_*ST*_ values (*F’*_*ST*_) were calculated in GenAlEx, assuming that each population has different alleles for each locus, which allowed us to estimate the maximum possible value of differentiation (Hedrick, 2005). To visualise a possible pattern of genetic structure, a PCoA was performed in GenAlEx with the obtained *F*_*ST*_ values.

To determine whether the genetic structure was driven by population distribution and geographic distance, we quantified IBD as the correlation between linearised *F*_*ST*_ (*F*_*ST*_/1- *F*_*ST*_) and log-transformed geographic distance in kilometres using a Mantel test (Mantel, 1967) with 10,000 permutations in GenAlEx. Geographic distance was calculated as the shortest path between sampling locations across the sea. These analyses were also performed independently for the populations in the three Mediterranean sub-basins (western and eastern Mediterranean and the Adriatic Sea). Furthermore, to identify potential barriers to gene flow for *C. caespitosa*, such as oceanographic fronts or currents, pairwise *F*_*ST*_ values and population geographic coordinates were included in the analyses performed in BARRIER v2.2 (Manni et al., 2004). To calculate barrier robustness, we generated 100 resampled bootstrap matrices in R (R Development Core Team, 2017), using an R script provided by Eric Petit (UMR ECOBIO CNRS, Paimpont, France).

Population genetic structure was assessed by a Bayesian clustering approach performed in STRUCTURE 2.3.4 (Pritchard et al., 2000). This program calculates population allele frequencies and, on the basis of HWE estimates, the probability of an individual belonging to one of the obtained genetic clusters. As we assumed that individuals can have ancestors from different locations, an admixture model was implemented with correlated allele frequencies and location as a prior. The analysis was run with a burn-in of 10,000 iterations and 100,000 Markov chain Monte Carlo, with a putative *K* of up to 25 (six clusters more than the number of sampling sites considered in the analysis) and 20 replicate runs. To infer the number of genetic clusters (best value of *K*), we applied three methods. We first used STRUCTURE HARVESTER (Earl and vonHoldt, 2012) and CLUMPAK (Kopelman et al., 2015) to evaluate the optimal *K* value following the method proposed by Evanno et al. (2005) to calculate Δ*K*. CLUMPAK was also employed to identify the *K* for which Pr (*K* = *k*) is the highest using ln (Pr (*X*/*K*)). Although the Evanno Δ*K* method may efficiently capture the uppermost level of structure, it may underestimate the number of groups (*K*) (Puechmaille, 2016). Therefore, we also estimated the number of clusters with the method of Puechmaille (2016) using StructureSelector (Li and Liu, 2018). We used the CLUMPAK web server to find the best alignment of the STRUCTURE results across the 20 replicates for each *K*.

The R package *adegenet* v2.1.1 (Jombart, 2008) was used to perform the DAPC. This method divides genetic variability of variance between and within groups and then optimises the variance between groups and minimises it within groups (Jombart, 2012), without considering whether population are in HWE.

To quantify the molecular variance, we ran the AMOVA in ARLEQUIN v3.11 (Excoffier et al., 2005) with 1000 permutations for all the populations and with the groups inferred by the STRUCTURE analyses. Putative first generation migrants were identified with a Bayesian assignment method (Rannala and Mountain, 1997) in GENECLASS2 (Piry et al., 2004).

Finally, we tested for recent reductions in effective population size using the allele frequency data in BOTTLENECK v1.2.02 (Cornuet and Luikart, 1996). Simulations were run under three mutation models: the infinite alleles model (IAM), the stepwise mutation model (SMM) and the two-phase mutation model (TPM) (Di Rienzo et al., 1994), with 10,000 iterations and a descriptor of allele frequency distribution (‘mode-shift’). Two statistical tests, the Sign test (Cornuet and Luikart, 1996) and the Wilcoxon sign-rank test (Luikart et al., 1998), were used to test each model.

## Results

Of the 19 microsatellites used in this study, one (L2) was discarded due to the ambiguity of its results. All of the analysed loci were polymorphic for all populations except L29, which was monomorphic for three of them (LIO, TOG and MEN), and V46 (for LIO).

Null alleles were detected in the populations analysed. As the presence of null alleles is known to inflate measures of genetic differentiation and cause overestimations of *F*_*ST*_ (Chapuis and Estoup, 2007), we repeated the population differentiation analyses correcting for the null alleles. However, no significant differences were found between the pairwise *F*_*ST*_ and the corrected *F*_*ST*_ values; therefore, we decided not to consider the null alleles correction.

### Clonal Structure in *C. caespitosa*

Of the 541 individuals genotyped, 482 (89%) had unique multilocus genotypes (*N*_*g*_) (Table 2). A total of 59 ramets belonging to 25 genets was detected. Clonality percentages of the populations varied from 0% (CDP, COL, PAL, OTR, TOG, TRE, POR and NEA) to 68.75% in CRE. Genotypic evenness (*G*_*o*_ */N*_*g*_) ranged from 0.21 for LIO to one for CDP, COL, PAL, OTR, TOG, TRE, POR and NEA. The lowest values of genotypic richness (*N*_*g*_ */N*) were detected for CRE and LIO. Even though these two populations, which have more clonal individuals than the others, had similar values of genetic richness, the observed genotypic diversity (*G*_*o*_) of LIO was half that of CRE, reflecting a difference in genet size (LIO had fewer but bigger genets). Genotypic diversity (*G*_*o*_ */G*_*e*_) varied from 0.08 for LIO, indicating “mostly asexual” reproduction (also for CRE and MEN), to one for CDP, COL, PAL, OTR, TOG, TRE, POR and NEA, indicating “sexual reproduction”. The others populations presented “mostly sexual” reproduction by the same criterion. The population with the lowest *D* index value was LIO, agreeing with the results obtained for genotypic diversity and genotypic richness. Due to the low number of unique multilocus genotypes detected for CRE (*N*_*g*_ = 5), we excluded this population from further analysis.

**TABLE 2:** Genotypic diversity of the analysed populations of *C. caespitosa* based on 19 microsatellites. *N*, number of polyps (colonies) sampled; *N*_*g*_, number of unique multilocus genotypes per site; *N*_*g*_ */N*, genotypic richness; *G*_*o*_, observed genotypic diversity; *G*_*o*_ */N*_*g*_ *,* genotypic evenness; *G*_*e*_, expected genotypic diversity(*N*); *G*_*o*_ */G*_*e*_, genotypic diversity and the *D* index.

Pop	*N*	*N*_*g*_	*N*_*g*_ */N*	*G*_*o*_	*G*_*o*_ */N*_*g*_	*G*_*e*_	*G*_*o*_ */G*_*e*_	*D*
CDP	30	30	1	30	1	30	1	1
GAV	20	19	0.95	18.18	0.96	20	0.91	0.99
ESP	31	28	0.90	24.64	0.88	31	0.80	0.99
COL	31	31	1	31	1	31	1	1
PUN	30	26	0.87	23.68	0.91	30	0.79	0.99
MEN	21	13	0.62	6.58	0.51	21	0.31	0.89
PAL	18	18	1	18	1	18	1	1
BON	29	27	0.93	25.48	0.94	29	0.88	0.99
POC	35	31	0.89	26.06	0.84	35	0.75	0.99
OTR	35	35	1	35	1	35	1	1
TOG	35	35	1	35	1	35	1	1
TRE	35	35	1	35	1	35	1	1
POR	14	14	1	14	1	14	1	1
KOR	35	34	0.97	33.10	0.97	35	0.95	0.99
BOK	34	33	0.97	32.11	0.97	34	0.94	0.99
CRE	16	5	0.31	4.13	0.82	16	0.26	0.81
NEA	31	31	1	31	1	31	1	1
LIO	31	11	0.35	2.33	0.21	31	0.08	0.58
KRY	30	26	0.87	22.50	0.86	30	0.75	0.98

### Genetic Variability in *C. caespitosa*

Linkage disequilibrium (LD) among loci was detected in some populations (in KRY for all loci, and in LIO, KOR, POC, COL, POR, TOG and OTR for some of them). Nevertheless, LD was not observed in any of the loci for all the populations; therefore, all loci were considered independent.

Standardised allelic richness (*N*_*a*_) across loci for each population ranged from 3.167 for LIO to 4.859 for NEA (Table 3). The mean value across all loci and populations was 4.412. Observed heterozygosity (*H*_*o*_) ranged from 0.477 in COL to 0.587 in LIO. Only LIO presented a *H*_*o*_ value greater than expected (*H*_*e*_) (0.587 and 0.496, respectively) and, therefore, a negative value for *F*_*IS*_ (-0.195), indicating a heterozygote excess. The rest of the populations presented lower values of *H*_*o*_ than *H*_*e*_ and positive *F*_*IS*_ values ranging from 0.042 in GAV to 0.16 in BON, indicating a heterozygosity deficit (or a homozygosity excess) and a high degree of inbreeding. All populations, except GAV and MEN, deviated from Hardy-Weinberg equilibrium (HWE). Effective population size estimates were relatively low, especially for MEN (*N*_*e*_ = 4.9), LIO (*N*_*e*_ = 0.4) and KRY (*N*_*e*_ = 4.9). In contrast, BON, TOG, KOR and NEA showed a theoretical infinite value.

**TABLE 3:** Genetic diversity and effective population size estimates for the 18 analysed populations of *C. caespitosa*. *N*_*a*_, standardised number of alleles; *H*_*o*_, observed heterozygosity; *H*_*e*_, expected heterozygosity; *F*_*IS*_, inbreeding coefficient; *N*_*e*_, effective population size. *populations that are not in HWE.

Pop	*N*_*a*_	*H*_*o*_	*H*_*e*_	*F*_*IS*_	*N*_*e*_
CDP	4.672	0.530	0.565	0.051*	319.9
GAV	4.278	0.538	0.563	0.042	233.9
ESP	4.242	0.501	0.563	0.118*	83.2
COL	4.232	0.477	0.533	0.097*	33.5
PUN	4.359	0.508	0.550	0.095*	96.6
MEN	4.056	0.509	0.524	0.043	4.9
PAL	4.561	0.532	0.599	0.118*	300.7
BON	4.697	0.532	0.597	0.161*	Infinite
POC	4.429	0.539	0.568	0.061*	19.8
OTR	4.758	0.507	0.542	0.069*	65.2
TOG	4.535	0.503	0.542	0.069*	Infinite
TRE	4.758	0.526	0.571	0.065*	110.7
POR	4.455	0.516	0.563	0.085*	15.0
KOR	4.616	0.532	0.587	0.088*	Infinite
BOK	4.621	0.506	0.559	0.101*	76.8
NEA	4.859	0.544	0.587	0.072*	Infinite
LIO	3.167	0.587	0.496	−0.195*	0.4
KRY	4.116	0.510	0.563	0.130*	4.9
Mean	4.412	0.522	0.560	0.072	—

### Population Differentiation in *C. caespitosa*

**FIGURE 2:**
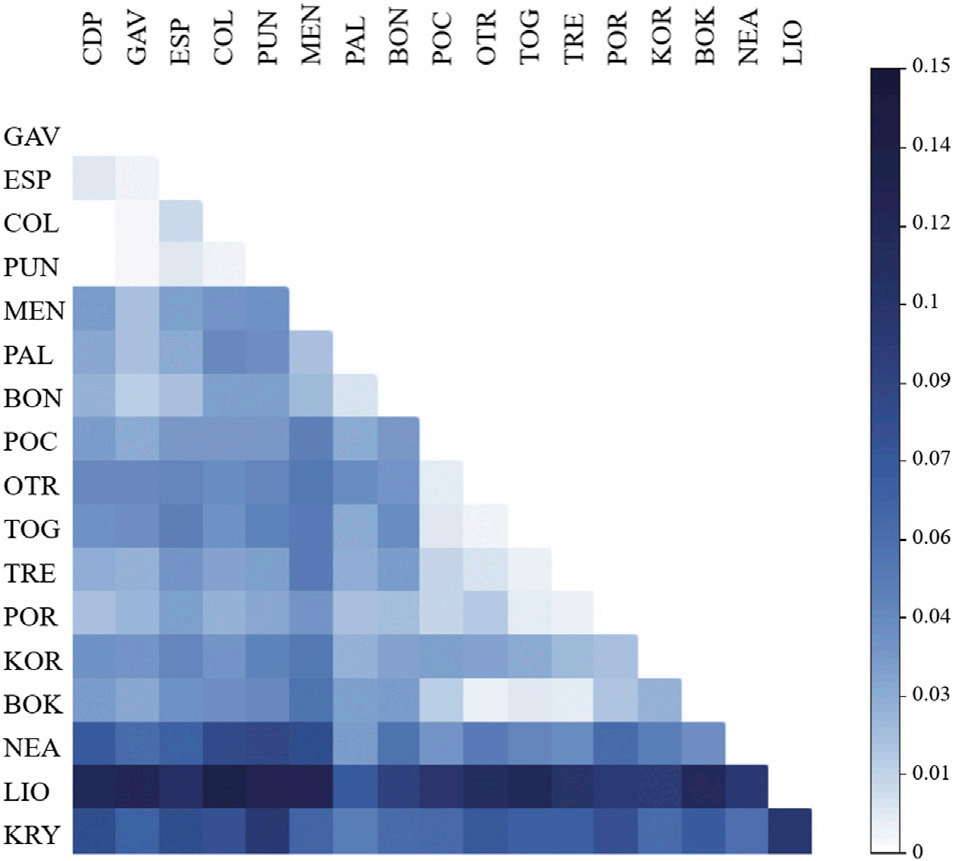
Matrix of pairwise *F*_*ST*_ comparisons between populations of *C. caespitosa*.

The global value of *F*_*ST*_ revealed a low but significant level of genetic differentiation (*F*_*ST*_ global = 0.043, *p* = 0). Pairwise *F*_*ST*_ values ranged from zero for CDP vs. GAV, CDP vs. COL and CDP vs. PUN (that is, among the western populations) to 0.134 for COL vs. LIO (i.e., between one western and one eastern population) (Figure 2; Table 4). The standardised *F*_*ST*_ value for COL vs. LIO was 0.279. Overall, three ranges of *F*_*ST*_ values were detected: from 0 to 0.02 (usually below 0.01) for within region comparisons, 0.02 to 0.06 for comparisons between populations from different regions and 0.06 to 0.13 for comparisons between any of the populations and the Cypriot ones (LIO and KRY), including between them. Notably, we observed relatively low *F*_*ST*_ values between the Greek population (NEA) and the Ionian (POC, *F*_*ST*_ = 0.039) or the Sardinian (PAL, *F*_*ST*_ = 0.037) ones, and high values between KOR and the other Adriatic populations.

**TABLE 4:** Pairwise *F*_*ST*_ values for the analysed populations of *C. caespitosa* (lower diagonal).

	CDP	GAV	ESP	COL	PUN	MEN	PAL	BON	POC	OTR	TOG	TRE	POR	KOR	BOK	NEA	LIO	KRY
CDP	**0**	0	0.019	0	0	0.083	0.075	0.059	0.086	0.103	0.1	0.071	0.051	0.099	0.105	0.174	0.260	0.198
GAV	0	**0**	0.008	0.005	0.005	0.051	0.052	0.038	0.075	0.101	0.103	0.069	0.062	0.096	0.095	0.147	0.263	0.162
ESP	**0.009**	0.0038	**0**	0.031	0.022	0.086	0.082	0.050	0.101	0.112	0.126	0.102	0.086	0.118	0.127	0.170	0.246	0.203
COL	0	0.0028	**0.013**	**0**	0.007	0.087	0.105	0.079	0.087	0.097	0.100	0.084	0.063	0.093	0.118	0.195	0.279	0.183
PUN	0	0.0028	0.009	0.003	**0**	0.095	0.102	0.085	0.094	0.108	0.117	0.090	0.075	0.117	0.133	0.209	0.271	0.236
MEN	**0.037**	**0.029**	**0.036**	**0.04**	**0.042**	**0**	0.054	0.059	0.120	0.121	0.127	0.127	0.096	0.129	0.151	0.197	0.261	0.158
PAL	**0.033**	**0.022**	**0.031**	**0.045**	**0.042**	**0.022**	**0**	0.027	0.080	0.103	0.076	0.074	0.055	0.067	0.098	0.094	0.169	0.134
BON	**0.027**	**0.017**	**0.021**	**0.035**	**0.035**	**0.024**	0.01	**0**	0.096	0.093	0.110	0.093	0.054	0.086	0.113	0.142	0.211	0.158
POC	**0.036**	**0.031**	**0.038**	**0.038**	**0.038**	**0.050**	**0.031**	**0.037**	**0**	0.011	0.027	0.036	0.036	0.080	0.039	0.093	0.231	0.151
OTR	**0.046**	**0.046**	**0.047**	**0.044**	**0.047**	**0.055**	**0.045**	**0.039**	0.006	**0**	0.006	0.018	0.041	0.075	0.016	0.123	0.243	0.172
TOG	**0.041**	**0.043**	**0.05**	**0.041**	**0.048**	**0.053**	**0.031**	**0.044**	**0.007**	0.004	**0**	0.009	0.021	0.073	0.032	0.116	0.264	0.170
TRE	**0.029**	**0.027**	**0.04**	**0.034**	**0.036**	**0.053**	**0.029**	**0.036**	**0.014**	**0.01**	0.005	**0**	0.016	0.059	0.027	0.109	0.239	0.172
POR	**0.022**	**0.027**	**0.035**	**0.028**	**0.032**	**0.04**	**0.022**	**0.023**	**0.014**	**0.02**	0.007	0.005	**0**	0.052	0.065	0.147	0.218	0.198
KOR	**0.042**	**0.04**	**0.047**	**0.04**	**0.048**	**0.054**	**0.028**	**0.033**	**0.034**	**0.034**	**0.03**	**0.024**	**0.022**	**0**	0.071	0.124	0.217	0.149
BOK	**0.037**	**0.032**	**0.041**	**0.042**	**0.045**	**0.058**	**0.036**	**0.037**	**0.017**	0.005	**0.008**	**0.007**	**0.02**	**0.028**	**0**	0.111	0.268	0.175
NEA	**0.074**	**0.062**	**0.068**	**0.084**	**0.088**	**0.083**	**0.037**	**0.058**	**0.039**	**0.053**	**0.047**	**0.043**	**0.062**	**0.051**	**0.042**	**0**	0.228	0.144
LIO	**0.12**	**0.122**	**0.110**	**0.134**	**0.126**	**0.124**	**0.074**	**0.092**	**0.102**	**0.114**	**0.12**	**0.105**	**0.099**	**0.097**	**0.118**	**0.1**	**0**	0.228
KRY	**0.083**	**0.067**	**0.083**	**0.08**	**0.099**	**0.067**	**0.052**	**0.062**	**0.064**	**0.076**	**0.07**	**0.07**	**0.08**	**0.063**	**0.073**	**0.059**	**0.101**	**0**

Significant values are in bold (*p* < 0.05). *F'*_*ST*_ values are shown in the upper diagonal.

**FIGURE 3:**
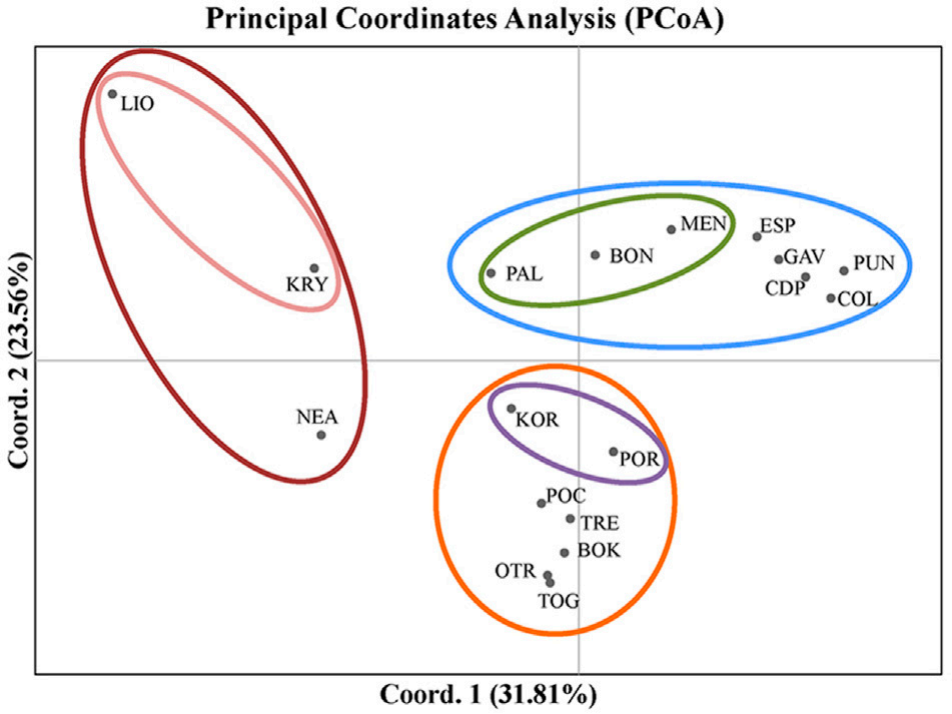
Results of the Principal Coordinates Analysis (PCoA) used to detect clustering of the populations of *C. caespitosa* on the basis of *F*_*ST*_ values. The first axis explains 31.8% of the variation, and the second, 23.56% of the variation.

The first two axes of the PCoA explained 55.36% of the variation in *F*_*ST*_ (Figure 3). In this analysis, the populations grouped according to the three main divisions of the Mediterranean basin (western, central and eastern). The western populations from the Spanish peninsular coasts (CDP, PUN and COL) grouped with those from the Balearic Formentera Islands (ESP and GAV). Populations located on the eastern side of the western sub-basin (PAL, BON and MEN) were also differentiated mainly by the first axis. The Adriatic populations, representing a distinctive sub-basin in the middle of the Mediterranean Sea, formed the second group, though with a clear separation between the northern populations of KOR and POR and the rest of the Adriatic populations. With respect to the eastern populations, LIO and KRY in Cyprus were separated from NEA in Greece by the second axis, though all three were clearly separated from one another.

To test whether an IBD pattern was present, we performed a Mantel test with the entire data set and with each of the three groups detected by the STRUCTURE analysis (see below). A pattern of IBD was only observed when all of the populations were analysed together (*R* ^*2*^ = 0.28; *p* = 0.001). No significant association between genetic differentiation (*F*_*ST*_) and geographic distance was observed when the populations were analysed separately according to sub-basin (Supplementary Figure S1).

**FIGURE 4:**
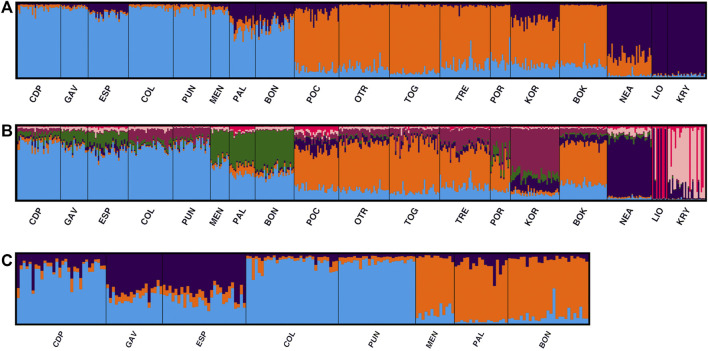
STRUCTURE results selected by Clumpak for the 18 analysed populations of *C. caespitosa* for **(A)** *K* = 3 and **(B)** *K* = 7. **(C)** Results for only the western Mediterranean populations for *K* = 3.

**FIGURE 5:**
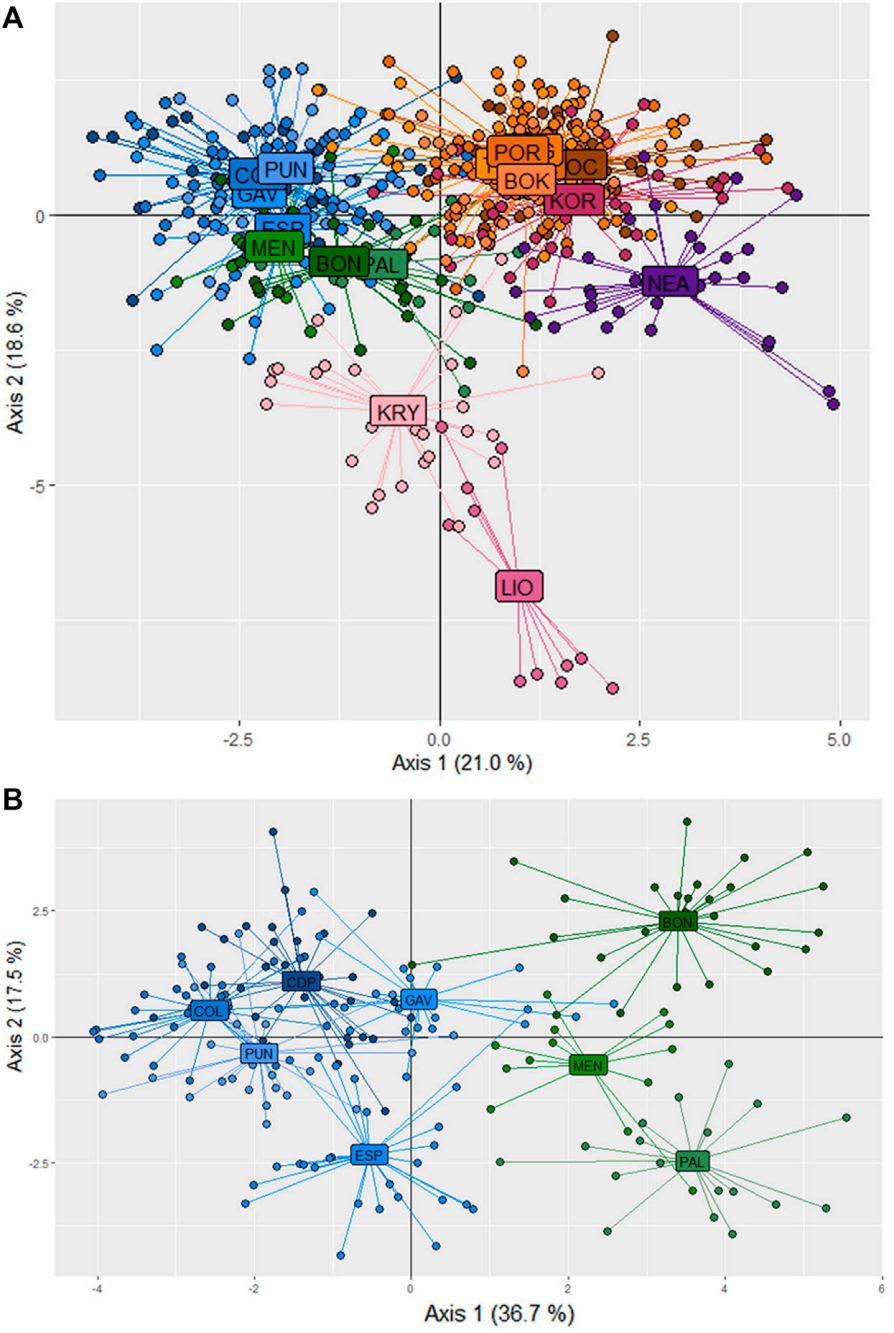
Population differentiation in *C. caespito*sa according to a DAPC analysis of **(A)** all populations or **(B)** only the western Mediterranean populations.

In the STRUCTURE analyses, three genetically differentiated clusters were detected with Evanno’s *K* method (Figure 4A). The results based on Pritchard’s method, which measures the probability (ln (Pr (X/*K*)), showed *K* = 9 as the best value. Under Puechmaille’s method, the populations of *C. caespitosa* were divided into seven clusters (Figure 4B). Results for *K* = 3 showed a clear division between the three Mediterranean sub-basins (western, central and eastern). The Tyrrhenian (PAL) and Ligurian (BON) populations showed a certain degree of admixture with Espardell Island (ESP) in Formentera. Other populations also showed admixture including KOR in the Adriatic Sea and NEA in the eastern Mediterranean. The results obtained with Puechmaille’s method (*K* = 7), though generally similar to those with Evanno’s method, showed a substructure within the basins. For instance, in the western Mediterranean, MEN, PAL and BON showed a high percentage of allocation to a particular cluster (besides the most frequent one in the western sub-basin). In addition, the singularity of KOR within the Adriatic Sea was evident, and LIO, NEA and KRY could each be distinguished as a separate group. When we considered *K* = 9, the results remain almost the same as those with a *K* = 7. Analysis of only the western populations revealed three sub-clusters: one differentiating MEN, PAL and BON; another differentiating COL and PUN and a third consisting of GAV and ESP, which are characterised by different degrees of admixture (Figure 4C). Cabo de Palos (CDP) showed similarities to both the COL and PUN and GAV and ESP sub-clusters. Supporting the general results of these analyses, the DAPC showed that the Adriatic and the western Mediterranean populations formed two separate groups, and that the Cypriot populations (LIO and KRY) and NEA were separate from each other and from the other groups (Figure 5A). Moreover, the DAPC of only the western individuals showed some distinction between the island localities MEN and PAL and the Ligurian one (BON) (Figure 5B).

The AMOVA of the three main groups detected by STRUCTURE (western, central and eastern Mediterranean) revealed that 94.67% of the genetic variation originated within populations, with only 2.84% of the variance being attributed to differences among groups. The lowest percentage of variation observed (2.49%) was among populations within groups. Similar results were obtained when seven groups were considered, with 94.75% of the genetic variation originating within populations (Table 5).

**TABLE 5:** AMOVA for the 18 analysed populations of *C. caespitosa* considering (A) three or (B) seven groups. *p* values for all of the results in both analyses were significant (*p* < 0.0001).

Source of Variation	d.f	Sum of Squares	Variance Components	Percentage of Variation
(A) Among groups	2	96.224	0.12891	2.84
Among populations within groups	15	153.617	0.11335	2.49
Within populations	936	4026.192	4.30149	94.67
Total	953	4276.034	4.54376	—
(B) Among groups	6	179.889	0.19975	4.40
Among populations within groups	11	69.953	0.03848	0.85
Within populations	936	4026.192	4.30149	94.75
Total	953	4276.034	4.53972	—

In the migration analysis, high rates of first generation migrants were detected (Table 6). The populations with the highest numbers of migrants were CDP (10 migrants), PUN (6), POC (9) and BOK (6). The most important sink populations were ESP (8) and KOR (6). Migrant flow was most important at an intraregional level, with new individuals coming from populations within the same basin. Given that migrant detection analysis using data comprised of low *F*_*ST*_ values may be imprecise, we used the STRUCTURE results for the assignment analyses (Supplementary Table S1). These results showed that the individuals from the Formentera Islands were, in part, assigned to one of the same genetic cluster as those from Menorca Island and the Italian populations (PAL and BON). The population from the Ionian Sea (POC) showed genetic admixture with all three Mediterranean sub-basins. In the Adriatic Sea, the northern populations TRE and POR were assigned to one of the same genetic clusters as KOR, the most differentiated location. Between the Cypriot populations, LIO showed putative migrants from KRY.

**TABLE 6:** First generation migrant test for *C. caespitosa.* For each population (see acronyms in Table 1), individuals are presented according to their sampling site in rows, and in columns, to their origin.

Origin																			
POP	CDP	GAV	ESP	COL	PUN	MEN	PAL	BON	POC	OTR	TOG	TRE	POR	KOR	BOK	NEA	LIO	KRY	Total
CDP	—	—	—	—	—	—	—	—	—	1	—	—	—	—	—	—	—	—	1
GAV	1	—	—	—	1	—	—	—	—	—	—	—	—	—	—	—	—	—	2
ESP	2	2	—	—	1	1	—	—	—	—	—	—	—	—	1	1	—	—	8
COL	2	—	—	—	1	1	—	1	—	—	—	—	—	—	—	—	—	—	5
PUN	—	—	—	1	—	—	—	1	1			1	—	—	—	—	—	—	4
MEN	2	1	—	—	—	—	—	1	—	—	1	—	—	—	—	—	—	—	5
PAL	—	—	—	—	1	—	—	1	—	—	—	—	—	1	—	—	—	—	3
BON	2	1	—	—	—	—	1	—	—	—	—	—	—	—	—	—	—	—	4
POC	—	—	—	—	—	—	—	—	—	—	1	1	—	1	1	—	—	—	4
OTR	—	—	—	—	—	—	—	—	2	—	—	—	—	1	1	—	—	—	4
TOG	—	—	—	—	—	1	—	—	—	1	—	1	—	—	—	1	—	—	4
TRE	—	—	—	1	1		—	—	1	—	—	—	1	—	1	—	—	—	5
POR	—	—	—	—	—	—	—	—	1	—	2	1		—	—	—	—	—	4
KOR	—	—	—	—	1	—	—	—	1	1		1	2	—	—	—	—	—	6
BOK	—	—	—	—	—	—	—	—	—	1	1		1	—	—	—	—	—	3
NEA	1	—	—	—	—	—	—	1	1	—	—	—	—	—	1	—	—	—	4
LIO	—	—	—	—	—	—	—	—	—	—	—	—	—	—	—	—	—	1	1
KRY	—	—	—	—	—	—	—	—	2	—	—	—	—	—	1	—	1	—	4
Total	10	4	—	2	6	3	1	5	9	4	5	5	4	3	6	2	1	1	

The results of the BARRIER analysis revealed six supported barriers to gene flow (bootstrap value >95) for *C. caespitosa* in the Mediterranean basin. In the western Mediterranean, two main barriers appear to be present: one separating the north-eastern Spanish populations (COL and PUN) from those in the Formentera Islands (GAV and ESP), and another separating ESP (Formentera) from the Menorca population (MEN) (corresponding to the Balearic Front and Mallorca Channel respectively, see Figure 1). A barrier also appears to separate the northernmost western populations (BON and PUN) from one another. A barrier that runs between Sicily and North Africa (Sicilian Channel) separates all of the western populations from the central and eastern ones (Figure 1). In the Adriatic Sea, a north-south barrier appears to divide the eastern coastal populations from the western ones, except in the north (for POR and TRE). Between the eastern and the central populations, a supported barrier was only observed between OTR and NEA (Aegean Front in Figure 1). Finally, in relation to population bottlenecks, only LIO displayed a mode shift, indicating it has experienced a recent bottleneck (Table 7).

**TABLE 7:** Bottleneck analysis results for the analysed populations of *C. caespitosa*.

	Sign Test			Wilcoxon Test			Mode Shift
	IAM	SMM	TPM	IAM	SMM	TPM	
CDP	0.23112	**0.00847**	0.53546	**0.02693**	0.98288	0.46614	Normal
GAV	**0.00251**	0.31129	0.05764	**0.01184**	0.63314	**0.04488**	Normal
ESP	**0.04321**	0.31652	0.47442	**0.03327**	0.90181	0.28992	Normal
COL	0.22302	**0.02989**	0.33052	0.23415	0.99088	0.73869	Normal
PUN	0.10110	**0.02814**	0.33920	**0.02693**	0.98075	0.43252	Normal
MEN	0.31287	0.40186	0.35531	0.06618	0.79812	0.51839	Normal
PAL	0.13159	0.53566	0.30376	**0.01184**	0.50000	0.07076	Normal
BON	0.05388	0.29483	0.51086	**0.01712**	0.86774	0.15190	Normal
POC	**0.03860**	0.07945	0.52037	**0.00912**	0.81539	0.14186	Normal
OTR	0.11330	**0.00793**	0.51491	0.14186	0.99088	0.61699	Normal
TOG	0.31066	**0.01393**	0.12158	**0.04919**	0.98474	0.64405	Normal
TRE	0.10957	**0.03099**	0.51748	**0.02414**	0.92298	0.41586	Normal
POR	0.46105	0.06859	0.18705	0.09819	0.95512	0.56748	Normal
KOR	0.12798	**0.00784**	0.51569	**0.04937**	0.97586	0.63314	Normal
BOK	0.28149	0.26926	0.51297	0.14186	0.89393	0.69527	Normal
NEA	0.14254	**0.00677**	0.30314	0.06487	0.99720	0.64906	Normal
LIO	**0.00052**	0.13288	0.09412	**0.00019**	**0.01248**	**0.00314**	**Shifted**
KRY	0.42720	0.13931	0.51549	**0.03327**	0.94581	0.50000	Normal

The two statistical tests, Sign and Wilcoxon sign-rank, were conducted under three mutation models: infinite alleles (IAM), stepwise mutation (SMM) and two-phase mutation (TPM). Significant values are in bold. Mode shift is also indicated.

## Discussion

### Genetic Variability

*Cladocora caespitosa* displays both sexual and asexual reproductive modes, and local populations display differences in the prevalence of these modes. Eight populations across the Mediterranean Sea (three western, four Adriatic and one eastern) appear to undergo sexual reproduction exclusively, as no evidence of clones was found. Sexual reproduction is also dominant in most of the other populations as only a low level of asexual reproduction was observed in them. These results are consistent with those of a previous study showing that sexual reproduction is predominant in *C. caespitosa* (Kružić et al., 2008). By contrast, asexual reproduction is dominant in CRE and LIO (>60% clones), and to a lesser extent, in MEN (38.1% clones). These values are comparable to the level of clonality shown by coral species that reproduce mostly by fragmentation, such as *Pavona clavus* or *Acropora valida* (Aranceta-Garza et al., 2012). The high rate of asexual reproduction in the LIO population was recently related to local natural or anthropogenic disturbances (López-Márquez et al., 2021). This phenomenon has also been observed in other scleractinian corals, such as in populations of *Pocillopora verrucosa* after tropical storms (Aranceta-Garza et al., 2012). Currently, we do not know the specific circumstances that have led to the high level of asexual reproduction in the CRE and MEN populations, though we hypothesise that they also experienced local disturbances that resulted in high mortality rates. In Crete, for instance, tourism has greatly impacted the coastlines (Tsilimigkas et al., 2020), which may contribute to the high level of asexual reproduction observed in *C. caespitosa* in this area. In Menorca, colonies are located in a sheltered place with a low swell (J. Templado, personal observation), thus bioerosion may be weakening these colonies, leading to polyp detachment. The small estimated effective size of MEN also suggests this locality may have been affected by environmental disturbances that could have triggered asexual reproduction in these colonies, as has been observed in other coral species (Lirman, 2000). Therefore, it is accepted the hypothesis that the clonal structure varies across its geographic range due to different disturbances affecting the sexual/asexual reproductive ratio.

Despite having a low level of genotypic diversity (*G*_*o*_ */G*_*e*_), the genetic diversity (*H*_*o*_) of MEN was similar to nearly all of the other analysed populations of *C. caespitosa*, and analogous to those reported for other tropical corals (Baums, 2008). This finding suggests that the level of sexual reproduction in this population is enough to maintain its diversity (Bengtsson, 2003). In concordance with other studies (Baums, 2008; Polato et al., 2010; Casado-Amezúa et al., 2012), we observed positive values for the inbreeding index, a heterozygosity deficit in most populations and deviation from HWE in all populations, except GAV and MEN. Deviation from HWE in marine invertebrate populations may be associated with their sexual reproductive mode. For instance, inbreeding is widespread in most marine benthic invertebrates with limited dispersal capabilities (Riesgo et al., 2016). In *C. caespitosa*, fertilisation takes place in the surrounding water of the broadcasting colonies without sexual selection; therefore, inbreeding is prevalent in this sessile species (Addison and Hart, 2005).

### Population Structure

Given the life history traits of *C. caespitosa*, such as its supposedly low dispersal capability and high self-recruitment rates (Kružić et al., 2008; López-Márquez et al., 2019; this study in which a rate greater than 80% was estimated), we hypothesised that the species would have a strong population structure with a high level of differentiation. In contrast, and rejecting this hypothesis, we found that the populations studied had, in general, relatively low *F*_*ST*_ values among some nearby populations, indicating a low level of differentiation. However, we did observe a regional structure in the western Mediterranean according to the *F*_*ST*_ values: populations from the peninsular coasts of Spain (CDP, PUN and COL) and the Balearic island of Formentera (GAV and ESP) formed a single group, whereas the Menorca island population (MEN) was genetically more similar to the populations in the Tyrrhenian (PAL) and Ligurian (BON) sub-basins (see Figure 2). This substructure is likely mediated by dispersal along the coastline and some resistance to larval movement in the open sea since lower *F*_*ST*_ values were observed between the coastal populations than between these and the island ones. Similar results were found in the Adriatic and adjacent Ionian populations (López-Márquez et al., 2019) and in the eastern Mediterranean populations (López-Márquez et al., 2021), where the gene flow resulted to be mainly driven by the shoreline configuration and sea surface current which likely enhanced the dispersal of planulae among populations. Genetic differentiation of the Croatian locality KOR from its neighbours in the Adriatic Sea was evidenced by the relatively high *F*_*ST*_ values observed among these populations. López-Márquez et al. (2019) showed the same results with a different set of markers and inferred that this was due to the influence of a north-south barrier. Although we also observed this latitudinal separation with our data set, the inclusion of POR, the northernmost Adriatic population, suggests this differentiation is not as strong as previously observed. The *F*_*ST*_ values shown between POR and the other Adriatic populations were not as high as those between KOR and these populations. The north-south differentiation observed in the Adriatic Sea is likely mediated by dispersion along the coastline and some dispersal events through subgyres that promote connectivity between the southern populations. Aside from its northern location, KOR stands out as a differentiated cluster. With respect to the eastern Mediterranean populations (KRY and LIO, in the Levantine Sea, and NEA, in the Aegean Sea), pairwise *F*_*ST*_ comparisons indicate that all are differentiated from one another; however, in the STRUCTURE analysis (for *K* = 3), they form a single cluster.

The population structure resulting from the various analyses (i.e., STRUCTURE, DAPC and PCoA) coincides with the three main divisions of the Mediterranean Sea (western, central and eastern Mediterranean). According to the Mantel test results, this is likely due to geographic distance (IBD) along the eastern–western distribution axis of the Mediterranean populations (see Supplementary Figure S1).

In addition to geographic distance, barriers to gene flow have likely influenced the current population structure of *C. caespitosa*. The barriers we identified largely coincide with those found in other population genetic studies (Pascual et al., 2017; Constantini et al., 2018). For this reason, the results suggesting the presence of long-distance first generation migrants in NEA and KRY were unexpected. One possible explanation could be the evolutionary convergence of alleles from different locations, which would lead to low pairwise differentiation and, consequently, indicate the presence of migrants. The fact that the standardised *F*_*ST*_ values between the eastern Mediterranean and Adriatic and Ionian populations are higher than for other pairwise comparisons supports this premise. Otherwise, anthropogenic dispersal should be taken into account (Radziejewska et al., 2006): human-mediated transport, for instance, through lithic anchors or ballast stones in Phoenician times could have also led to this surprising result.

Regarding the intra-regional substructure detected by the STRUCTURE and assignment analyses, gene flow is not mediated by distance, but rather by ocean currents and dispersal along the coastlines. Consistent with this, the Balearic Front (BF) and the Mallorca Channel (MC, Ruiz et al., 2009) appear to act as barriers dividing CDP and the two Formentera populations (GAV and ESP) from PUN and COL. However, evidence of migrants and a low to moderate level of genetic differentiation indicate that these barriers are permeable. The NBF is seasonal, and it depends on the strength of the Northern Current (Font et al., 1995). As such, during autumn and winter, its intensity decreases and the barrier is less strong. This event almost matches the spawning period of *C. caespitosa* in this region (Kersting et al., 2013b), which would allow for some level of gene flow between populations. Another ocean front of importance is the Northern Tyrrhenian Gyre (Poulain et al., 2012), which facilitates connectivity between the populations from the Tyrrhenian and Ligurian seas (PAL and BON, respectively). On the other hand, connectivity among the eastern populations seem to be influenced mainly by a pattern of isolation by environment, consistent with the results shown by López-Márquez et al. (2021).

Lastly, although we only obtained five unique multilocus genotypes for CRE, leading us to exclude it from further analysis, our results on this population’s genetic structure (Supplementary Figure S2) were potentially interesting. Greater sampling effort of this area, however, is needed to confirm this hypothesis.

As discussed here, various factors affect the genetic differentiation of the different populations of *C. caespitosa* in the Mediterranean. The population structure of this coral mainly appears to be correlated with the division of the main Mediterranean sub-basins, and the barriers between them, together with favoured dispersal along coastlines. Despite the low dispersal ability, high rate of self-recruitment and estimated low effective population sizes of *C. caespitosa*, the occasional dispersive events that seem to occur between regions are sufficient to maintain the species’ considerable genetic diversity. Under suitable conditions and habitat continuity without barriers to gene flow, connectivity between distant colonies of *C. caespitosa* could be high. In fact, the two localities on the peninsular coast of Spain (CDP and PUN), which are separated by 400 km, did not present any genetic differentiation (pairwise *F*_*ST*_ value = 0).

### Conservation Implications

Despite its importance in conservation policies, knowledge of the genetic diversity and connectivity patterns of species is often not considered by policymakers. This type of information is especially important for the conservation of endangered structural species. Our results on the genetic structure and population connectivity of *C. caespitosa*, whose populations are in alarming decline, highlight key aspects that should be included in the conservation decision making process for this species. These aspects include the relatively low dispersal ability and the high rate of self-recruitment of the species, the importance of sporadic dispersal events to maintain diversity and the level of clonality of some populations.

The regression of populations of *C. caespitosa* is still in progress (Rodolfo-Metalpa et al., 2005), and the high frequency of mortality events in this coral over the last decades possibly exceeds its recovery potential because of its low recruitment rates (Kersting et al., 2014a). Therefore, understanding the general pattern of connectivity among populations of this coral, particularly in light of its discontinuous distribution (as isolated colonies or, more rarely, as beds or banks) and low dispersal ability, becomes increasingly important. Known beds and banks of the coral should be considered as source populations from which larvae can be occasionally exported to other areas. Conversely, populations comprised of a few scattered colonies for which successful fertilisation may prove highly difficult can serve as potential recipient populations. For this reason, strict protection of the marine areas where beds and banks of *C. caespitosa* are known to be present should be a top priority. According to the available information from the literature and expert observations describing the abundance and morphology of colonies (compiled by Chafaoui et al., 2017), only 31 localities across the entire Mediterranean basin have reported bioherms of the species. Although in this study we cannot appraise whether the current level of protection in established marine protected areas is sufficient for the conservation of the species, we strongly recommend an increase in the coverage of this emblematic species within these areas and measures to preserve it from major threats, particularly those that are mediated by human activities. For instance, although extreme climatic events, such heat waves, appear to be a major threat to this coral (Kersting et al., 2013a; Jiménez et al., 2016), other factors posing a threat include the spread of mucilage and invasive algae (Kersting et al., 2014b), eutrophication due to sewage discharge or fish farming (Kružić and Požar-Domac, 2007), trawling, anchorage and high sedimentation rates caused by dredging or extreme storms (Casado de Amezua et al., 2015; Chefaoui et al., 2017; López-Marquez et al., 2021). In addition to the conservation of the known coral beds, prospecting studies of other Mediterranean localities should be conducted to identify as yet unknown populations of the coral.

Besides local impacts, global changes (sea warming and acidification) also threaten *C. caespitosa*. Global threats such heat waves or extreme storms interact with local ones such as pollution or coastal degradation, leading to cumulative impacts that are particularly high in coastal ecosystems (Micheli et al., 2013). Although local mitigation strategies cannot directly protect populations from the impact of global threats, they are often the only feasible way to reduce the synergy between the different types of threats, and to help preserve marine coastal ecosystems (Templado, 2014). In this context, protection against local impacts is of the utmost importance.

Another aspect to consider in the conservation of *C. caespitosa* is the role of asexual reproduction, and its effect on the genetic diversity of populations. For sessile organisms that undergo external fertilisation, asexual reproduction may be the local means of proliferation for populations with a small population size and/or low gamete density (“Allee effect”; Courchamp et al., 2008). Clonal growth, therefore, may allow the species to persist through periods of low sexual recruitment (Lasker and Coffroth, 1999); however, it may also lead to reduced genotypic diversity and, as a result, higher susceptibility to environmental changes (Reusch et al., 2005). From a conservation point of view, knowledge of the genetic and genotypic diversity patterns of structural species with a high potential for asexual reproduction is critical: populations with a high level of genotypic diversity may be better able to withstand environmental changes or extreme climatic events (Reusch et al., 2005) and, conversely, those with a low level of genetic diversity may be more vulnerable to pathogens and parasites (King and Lively, 2012). For *C. caespitosa*, we found that the ratio of clonal to sexual recruitment is highly variable between localities, and is likely a consequence of a complex interplay of various impacts. Future studies should focus on determining the factors that affect this ratio and how different impacts interact synergistically on it, as well as on the historical causes for the decline of bioherms of the species. Increased clonality may negatively impact the adaptive potential of the species under the current disturbance regime; therefore, it should be closely monitored.

In summary, enforcement of conservation measures that aim to protect the genetic diversity of endangered structural species such *C. caespitosa* is essential. The results provided here, combined with those from a recent study on another endangered Mediterranean coral, *Astroides calycularis* (Ledoux et al., 2021), may help build a highly relevant framework in which to study the evolution of Mediterranean marine diversity as it faces the increasing warming of the basin waters.

## Data Availability

The datasets presented in this study can be found in online repositories. The names of the repository/repositories and accession number(s) can be found in the article/Raw Data and Supplementary Material.
