# Supplementary material for: Genetic Structure of the Endangered Coral Cladocora caespitosa Matches the Main Bioregions of the Mediterranean Sea
Source: Front Genet. 2022 Jul 26;13:889672. doi: 10.3389/fgene.2022.889672 (PMC9360616; doi:10.3389/fgene.2022.889672)
Supplement: Supplementary file 2 [file DataSheet1.docx]

Supplementary Material

# Supplementary Figures and Tables

Supplementary Figure 1. Mantel test results (IBD) for A, all populations; B, Western Mediterranean populations; C, Adriatic and Ionian populations.

**
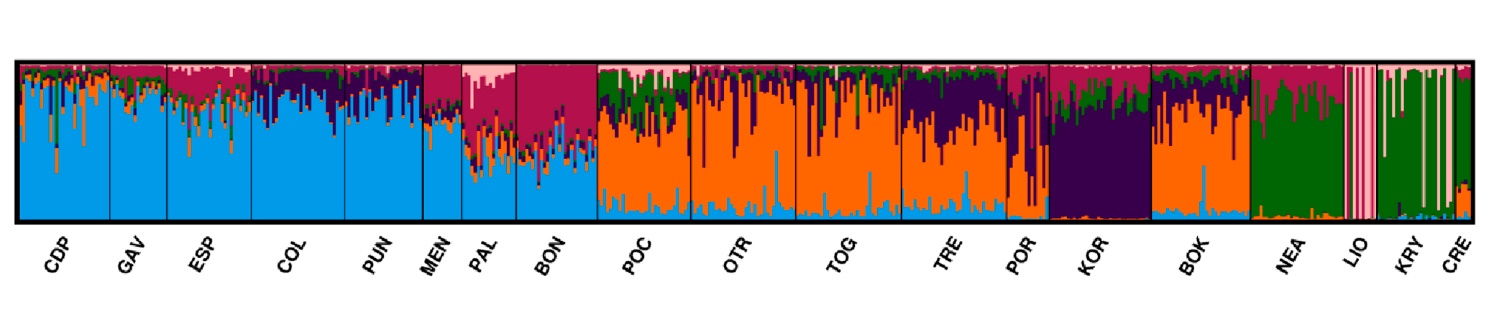
Supplementary Figure 2.** STRUCTURE results selected by Clumpak for the populations of Cladocora caespitosa including Crete (CRE) for K = 6.

**Supplementary Table 1.** Percentage assignment to each group of the STRUCTURE results for the 18 populations.

|  | Group 1 | Group 2 | Group 3 | Group 4 | Group 5 | Group 6 | Group 7 | N |
| --- | --- | --- | --- | --- | --- | --- | --- | --- |
| CDP | 0 | 3 | 1 | 3 | 3 | 80 | 5 | 30 |
| GAV | 0 | 10 | 2 | 3 | 1 | 78 | 3 | 19 |
| ESP | 2 | 11 | 5 | 5 | 1 | 72 | 2 | 28 |
| COL | 0 | 3 | 1 | 1 | 11 | 79 | 1 | 31 |
| PUN | 0 | 1 | 0 | 1 | 8 | 82 | 5 | 26 |
| MEN | 1 | 33 | 2 | 1 | 1 | 57 | 4 | 13 |
| PAL | 6 | 35 | 3 | 2 | 2 | 41 | 9 | 18 |
| BON | 0 | 48 | 3 | 2 | 1 | 40 | 5 | 27 |
| POC | 5 | 1 | 4 | 15 | 8 | 14 | 52 | 31 |
| OTR | 0 | 2 | 1 | 7 | 9 | 15 | 64 | 35 |
| TOG | 0 | 0 | 2 | 4 | 12 | 11 | 67 | 35 |
| TRE | 1 | 1 | 2 | 6 | 20 | 19 | 48 | 35 |
| POR | 0 | 10 | 0 | 4 | 32 | 13 | 38 | 14 |
| KOR | 0 | 9 | 3 | 9 | 61 | 8 | 7 | 34 |
| BOK | 1 | 1 | 2 | 8 | 8 | 19 | 59 | 33 |
| NEA | 2 | 7 | 15 | 69 | 1 | 2 | 3 | 31 |
| LIO | 55 | 0 | 8 | 36 | 0 | 0 | 0 | 11 |
| KRY | 19 | 0 | 75 | 1 | 0 | 1 | 0 | 26 |
